# Supplementary material for: A Systematic Review of Diagnostic Modalities and Strategies for the Assessment of Complications in Adult Patients with Neurofibromatosis Type 1
Source: Cancers (Basel). 2024 Mar 11;16(6):1119. doi: 10.3390/cancers16061119 (PMC10968786; doi:10.3390/cancers16061119)

A systematic review of international adult Neurofibromatosis 1 surveillance

Supplementary Table S1: Search strategy
Supplementary Table S2: Quality assessment of included cohort studies using Joanna Brigg's Institute Critical Appraisal tool.
Supplementary Figure S1: Common clinical manifestations in adult NF1 patients

Supplementary Table S1: Search strategy

PubMed	
**#1**	NF1 [Title/Abstract] OR “Neurofibromatosis 1” [Title/Abstract]OR “Neurofibromatosis Type 1” [Title/Abstract]OR “Neurofibromatosis Type I” [Title/Abstract] OR "Genes, Neurofibromatosis 1"[Mesh]
**#2**	Surveill* [Title/Abstract] OR Monitor* [Title/Abstract] OR Scan* [Title/Abstract] OR Tomography [Title/Abstract] OR MRI [Title/Abstract] OR CT [Title/Abstract] OR PET [Title/Abstract] OR Imag* [Title/Abstract]OR Pressure [Title/Abstract] OR “Physical examination” [Title/Abstract] OR “Clinical examination” [Title/Abstract] OR "Magnetic Resonance Imaging"[Mesh] OR "Positron Emission Tomography Computed Tomography"[Mesh] OR "Positron-Emission Tomography"[Mesh] OR "Tomography, X-Ray Computed"[Mesh] OR "Blood Pressure Determination"[Mesh] OR "Physical Examination"[Mesh]
**#3**	"Adult"[Mesh] OR "18 years"[Title/Abstract:~3] OR "aged 18"[Title/Abstract:~3] OR "18 and above"[Title/Abstract:~3]
**Embase**	
**#1**	nf1:ti,ab OR 'neurofibromatosis 1':ti,ab OR 'neurofibromatosis type 1':ti,ab OR 'neurofibromatosis Type I':ti,ab OR 'neurofibromatosis type 1'/exp
**#2**	surveill*:ti,ab OR monitor*:ti,ab OR scan*:ti,ab OR tomography:ti,ab OR MRI:ti,ab OR Imag*:ti,ab OR PET:ti,ab OR CT:ti,ab OR 'pressure NEAR/5 measurement':ti,ab OR 'clinical exam*':ti,ab OR 'physical exam*':ti,ab OR 'monitoring'/exp OR 'nuclear magnetic resonance imaging'/exp OR 'positron emission tomography'/exp OR 'computer assisted tomography'/exp OR 'PET-CT scanner'/exp OR 'blood pressure measurement'/exp OR 'clinical examination'/exp
**#3**	'young adult'/exp OR 'middle aged'/exp OR 'aged'/exp OR '18 NEAR/3 years':ti,ab OR 'aged NEAR/3 18':ti,ab OR '18 NEAR/3 above 18':ti,ab

Supplementary Table S2: Quality assessment of included cohort studies using the Joanna Briggs Institute critical appraisal tool

Study	Year	1	2	3	4	5	6	7	8	9	10	11
Avanesov	2021	Y	Y	Y	Y	Y	N	Y	NA	NA	NA	Y
Brahmi	2015	Y	Y	Y	Y	Y	N	Y	Y	Y	N	Y
Brenner	2006	Y	Y	Y	Y	Y	N	Y	Y	Y	N	Y
Chirindel	2015	Y	Y	Y	U	U	N	Y	Y	Y	N	Y
Cook	2017	Y	Y	Y	Y	Y	N	Y	NA	NA	NA	Y
Heervä	2013	Y	Y	Y	Y	Y	N	Y	Y	Y	Y	Y
Salamon 2013	2013	Y	Y	Y	Y	Y	N	Y	Y	Y	N	Y
Salamon 2015	2015	Y	Y	Y	Y	Y	N	Y	NA	NA	NA	Y
Salamon 2014	2014	Y	Y	Y	Y	Y	N	Y	Y	Y	N	Y
Seitz	2010	Y	Y	Y	Y	Y	Y	Y	Y	Y	Y	Y
Van Der Gucht	2016	Y	Y	Y	Y	Y	N	Y	NA	NA	NA	Y
Bredella	2007	Y	Y	Y	U	U	N	Y	NA	NA	NA	Y
Debnam	2014	Y	Y	Y	Y	Y	N	Y	NA	NA	NA	Y

Modica	2023	Y	Y	Y	Y	Y	N	Y	NA	NA	NA	Y
Nishida 2016	2016	Y	Y	Y	Y	Y	N	Y	Y	Y	N	Y
Nishida 2021	2021	Y	Y	Y	Y	Y	N	Y	Y	Y	N	Y
Combemale	2014	Y	Y	Y	Y	Y	N	Y	Y	Y	Y	Y
Arigon	2002	Y	Y	Y	Y	Y	N	Y	NA	NA	NA	Y
Khosrotehrani	2003	Y	Y	Y	U	U	N	Y	Y	Y	N	Y
Ahlawat	2019	Y	Y	Y	Y	Y	N	Y	Y	Y	N	Y
Alkan	2005	Y	Y	Y	Y	Y	N	Y	NA	NA	NA	Y
Cai	2009	Y	Y	Y	Y	Y	N	Y	NA	NA	NA	Y
Derlin	2003	Y	Y	Y	U	U	N	Y	Y	Y	Y	Y
Feldmann	2003	Y	Y	Y	Y	Y	N	Y	NA	NA	NA	Y
Ferner	2000	Y	Y	Y	Y	Y	N	Y	Y	Y	Y	Y
Friedrich	2005	Y	Y	Y	U	U	N	Y	NA	NA	NA	Y
Mautner	2006	Y	Y	Y	U	U	N	Y	NA	NA	NA	Y
Sellmer	2017	Y	Y	Y	Y	Y	N	Y	Y	Y	Y	Y

Jaremko	2012	Y	Y	Y	Y	Y	N	Y	NA	NA	NA	Y
Koike	2022	Y	Y	Y	Y	Y	N	Y	NA	NA	NA	Y
Matsumine	2009	Y	Y	Y	Y	Y	N	Y	NA	NA	NA	Y
Afridi	2015	Y	Y	Y	Y	Y	N	Y	NA	NA	NA	Y
Chhabra	2011	Y	Y	Y	Y	Y	N	Y	NA	NA	NA	Y
Sellmer	2018	Y	Y	Y	Y	Y	N	Y	Y	Y	N	Y
Broski	2016	Y	Y	Y	Y	Y	N	Y	Y	Y	N	Y
Curtis-Lopez	2020	Y	Y	Y	Y	Y	Y	Y	NA	NA	NA	Y
Sheerin	2022	Y	Y	Y	Y	Y	N	Y	NA	NA	NA	Y
Salamon 2019	2019	Y	Y	Y	Y	Y	N	Y	NA	NA	NA	Y
Ramachandran	2004	Y	Y	Y	Y	Y	N	Y	Y	Y	Y	Y
Pecoraro	2017	Y	Y	Y	Y	Y	Y	Y	NA	NA	NA	Y
Heffler	2017	Y	Y	Y	Y	Y	N	Y	NA	NA	NA	Y
Tucker	2005	Y	Y	Y	Y	Y	N	Y	NA	NA	NA	Y
Ina Ly	2023	Y	Y	Y	Y	Y	N	Y	Y	Y	N	Y

Well	2020	Y	Y	Y	Y	Y	N	Y	NA	NA	NA	Y
Van Meerbeeck	2009	Y	Y	Y	Y	Y	N	Y	NA	NA	NA	Y
Well	2021	Y	Y	Y	Y	Y	N	Y	NA	NA	NA	Y
Mautner	2008	Y	Y	Y	Y	Y	N	Y	NA	NA	NA	Y
Plotkin	2012	Y	Y	Y	Y	Y	N	Y	NA	NA	NA	Y
Zhang	2017	Y	Y	Y	Y	Y	N	Y	NA	NA	NA	Y
Byrne	2017	Y	Y	Y	Y	Y	N	Y	Y	Y	Y	Y
Guillamo	2003	Y	Y	Y	Y	Y	N	Y	Y	Y	Y	Y

Checklist
1. Were the two groups similar and recruited from the same population?
2. Were the exposures measured similarly to assign people to both exposed and unexposed groups?
3. Was the exposure measured in a valid and reliable way?
4. Were confounding factors identified?
5. Were strategies to deal with confounding factors stated?
6. Were the groups/participants free of the outcome at the start of the study (or at the moment of exposure)?
7. Were the outcomes measured in a valid and reliable way?
8. Was the follow up time reported and sufficient to be long enough for outcomes to occur?
9. Was follow up complete, and if not, were the reasons to loss to follow up described and explored?
10. Were strategies to address incomplete follow up utilized?
11. Was appropriate statistical analysis used?

Legend:

Y – Yes

N – No

U – Unclear

NA – Not applicable

Supplementary Figure S1: Common clinical manifestations in adult NF1 patients

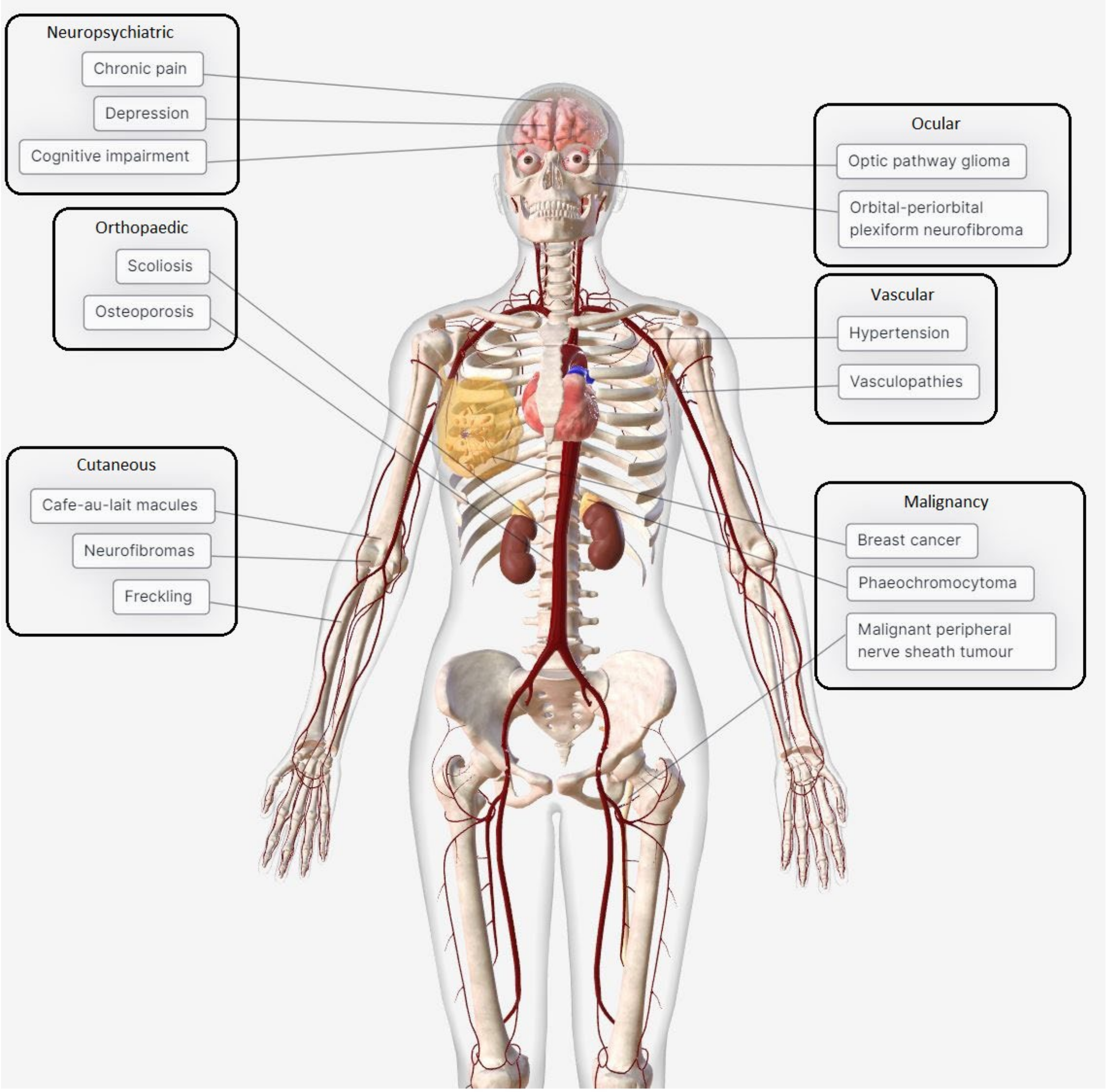

Supplement: Supplementary file 1 [file cancers-16-01119-s001.zip › cancers-2873616-supplementary.pdf]
